# Trophic Diversity in Duckweed: Mixotrophy, More Than the Sum of its Extremes

**DOI:** 10.1002/advs.75665

**Published:** 2026-05-14

**Authors:** Zuoliang Sun, Yan Chen, Fajun Li, Xuyao Zhao, Qingxiang Han, Hongwei Hou

**Affiliations:** ^1^ Jia Sixie College of Agriculture Shandong Provincial University Laboratory for Protected Horticulture Weifang University of Science and Technology Shouguang P. R. China; ^2^ Special Agricultural Resources in Tuojiang River Basin Sharing and Service Platform of Sichuan Province Analysis and Testing Center College of Chemistry and Chemical Engineering Neijiang Normal University Neijiang Sichuan P. R. China; ^3^ State Key Laboratory of Breeding Biotechnology and Sustainable Aquaculture Institute of Hydrobiology Chinese Academy of Sciences Wuhan Hubei P. R. China; ^4^ Institute of Emerging Agricultural Technology Shenzhen University of Advanced Technology Shenzhen P. R. China; ^5^ Aquatic Plant Research Center Wuhan Botanical Garden Chinese Academy of Sciences Wuhan P. R. China

**Keywords:** aquatic plant, carbon cycling, duckweed, heterotrophy, mixotrophy, synergistic effect

## Abstract

The duckweed family Lemnaceae, aquatic plants within the early‐diverging monocot order Alismatales, are model organisms in plant biology. Owing to their exceptionally rapid growth rates, high protein content, notable capacity for starch accumulation, and demonstrated utility in phytoremediation, duckweeds are increasingly recognized as promising candidates for diverse biotechnological applications spanning environmental remediation, bioenergy production, and sustainable biomass generation. Mixotrophy‐the capacity of autotrophic plants to utilize exogenous organic carbon‐is increasingly recognized as widespread in aquatic ecosystems, yet it has been less studied in duckweeds compared to microalgae and cyanobacteria. Here, we compile evidence from physiological, structural, and environmental perspectives supporting mixotrophy and heterotrophy in duckweeds, a clonal, genomically tractable angiosperm with rapid vegetative growth and direct environmental exchange, offering advantages beyond microalgal models. We emphasize mechanisms that enable mixotrophy to achieve synergistic outcomes beyond the additive effects of autotrophy and heterotrophy. Additionally, we explore the ecological and biotechnological significance of these trophic strategies and propose that duckweeds represent a valuable model for understanding mixotrophy across aquatic plants and for linking microalgal findings with higher‐plant physiology.

## Graphical abstract

In the context of rising DOC in aquatic environments, mixotrophic duckweed may impact carbon cycling by acting as either a carbon sink, as they absorb CO_2_ through photosynthesis, or a carbon source, as they release CO_2_ through respiration of absorbed DOC, which depends on DOC concentration, light availability, temperature, and other environmental factors.

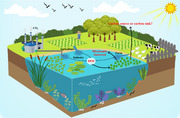

## 1 Introduction

Carbon acquisition in living organisms has often been described along a continuum bounded by photoautotrophy and heterotrophy, although increasing evidence now shows that these strategies can coexist within the same organism or even within the same cell under certain conditions [1]. This dichotomy is historically rooted in epistemological reasoning, as macroscopic organisms‐readily observable and often used as research models‐appear to conform to this segregation. Consequently, trophic webs are typically described in terms of opposing groups: primary producers, which generate primary biomass, and consumers, which produce secondary or higher‐level biomass [2].

However, growing evidence demonstrates that mixotrophy is pervasive in both aquatic and terrestrial ecosystems [2, 3, 4, 5]. While awareness of mixotrophy in land plants has increased, reports in aquatic higher plants remain limited and fragmented [3]. Moreover, terrestrial and aquatic research groups often investigate distinct taxa and ecosystems, which hinders recognition of the ubiquity of mixotrophy across the biosphere [1]. In addition, the marked differences between aquatic and terrestrial environments have reinforced the underestimation of shared traits and questions, yet mixotrophy exemplifies a unifying mechanism suggesting convergent constraints across environments [3, 4]. This highlights the need for a conceptual framework to understand mixotrophy across eukaryotic lineages and ecosystems.

Duckweeds are aquatic monocotyledonous plants with rapid vegetative propagation and high starch yields, representing both ecological and economic significance [6]. Remarkably, duckweed demonstrates not only mixotrophy but also heterotrophy and photoheterotrophy traits long overlooked due to the prevailing paradigm that plants are strict autotrophs [5]. In this review, we examine phylogenetic and ecological evidence for mixotrophy in duckweeds, emphasizing underlying mechanisms. We highlight the diverse physiological processes enabling mixotrophy and assess their ecological implications. Furthermore, we propose a unifying hypothesis that mixotrophy is a recurrently selected strategy in aquatic ecosystems, reflecting ecosystem dynamics and evolutionary drivers.

## 2 Duckweeds are Useful for Fundamental Research and Commercial Applications

Duckweeds (family Lemnaceae), a group of aquatic plants with C_3_ photosynthesis, comprise five genera (*Spirodela*, *Landoltia*, *Lemna*, *Wolffiella*, and *Wolffia*) encompassing 36 species [7, 8], though recent investigations suggest 35 species and at least three well‐described hybrids [9]. They are highly productive in terms of biomass yield and are widely distributed from temperate to tropical regions, commonly occurring in freshwater ponds, ditches, and other still or slow‐moving waters [10]. These minute plants (1 mm to ∼1.5 cm) consist of small fronds and adventitious roots, making them excellent systems for studying root development and evolution [6, 11]. Duckweeds primarily propagate asexually and can double their biomass in ∼16–48 h under optimal conditions [12]. Owing to their rapid growth and high protein content (35–40% of dry weight), duckweeds have long served as food and animal feed, particularly in Southeast Asia [13]. However, their entry into broader food and feed markets is currently hampered by strict regulations, such as those imposed by EFSA (European Food Safety Authority). Under various stress conditions, duckweeds accumulate large quantities of starch granules in their chloroplasts (75% dry weight) [14]. Their low cellulose (10% dry weight) and lignin (2% dry weight) contents also facilitate efficient conversion to bioethanol via fermentation [15]. Moreover, crude duckweed extracts exhibit antimicrobial activity against waterborne bacteria and fungi [16, 17], suggesting potential therapeutic applications. Overall, duckweeds represent a valuable resource for both fundamental research and industrial biotechnology [6].

## 3 Favorable Aquatic Environments for Duckweeds With Mixotrophic and Heterotrophic Abilities

Duckweeds evolved from terrestrial ancestors that secondarily colonized freshwater ecosystems [11]. They provide a variety of ecosystem services, including shelter and food for macroinvertebrates and microorganisms [18, 19]. As major primary producers, they contribute substantially to aquatic food webs and help moderate high water temperatures by providing shade during summer [20]. Moreover, duckweeds efficiently absorb excess nutrients and harmful elements such as nitrogen, phosphorus, heavy metals, and organic pollutants, thereby improving water quality and restoring ecological function in aquatic environments [6]. Their significant resistance to various environmental contaminants and pollutants also makes them convenient bioindicators of water pollution [6].

**FIGURE 1:**
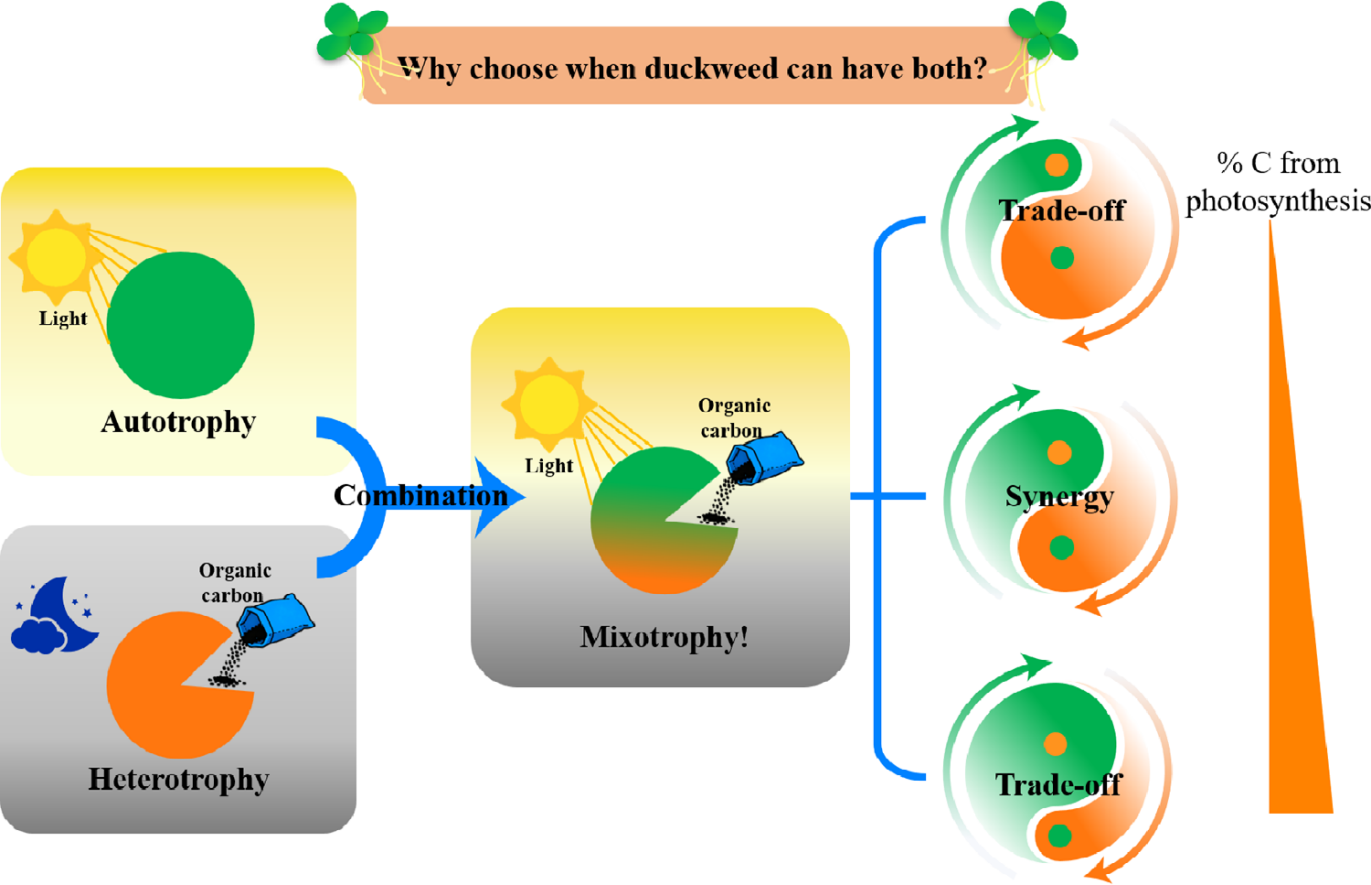
Photoautotrophy and heterotrophy are integrated in mixotrophy, which provides access to additional carbon sources and compensates for reduced photosynthesis under low‐light conditions. In mixotrophy, exogenous carbon and light are supplied concurrently, and both photoautotrophic and heterotrophic metabolisms operate within a single cell. Mixotrophy thus represents a functional continuum between strict photoautotrophy and strict heterotrophy. As the reliance on heterotrophic nutrition increases, the ratio of carbon fixed by photosynthesis within mixotrophic biomass decreases.

Despite these recognized ecological roles, an important aspect of duckweed metabolism has only recently gained attention. Duckweeds were conventionally regarded as strictly photoautotrophic [3]. However, recent findings show that they can also perform mixotrophic, heterotrophic, and even photoheterotrophic growth (uses light but relies on external organic C) [5]. Comparable strategies are observed in terrestrial (*Cuscuta australis*) and aquatic (*Utricularia gibba*) plants that partially rely on heterotrophic nutrition [21, 22], indicating that such plants simultaneously utilize photosynthetically fixed carbon and exogenous organic matter‐a phenomenon defined as mixotrophy (Figure 1) [23]. Depending on the trade‐off or synergy between autotrophy and heterotrophy, the proportion of biomass carbon (C) derived from photosynthesis in duckweed can vary substantially (Figure 1).

Freshwater ecosystems are rich in dissolved organic carbon (DOC), derived from the decomposition of plant and animal residues, and are increasingly contaminated by organic carbon‐rich wastewater from industrial and agricultural activities [24]. Duckweeds frequently experience light limitation due to shading by surrounding vegetation or debris, conditions that severely constrain their photosynthetic potential [25]. Additionally, the night period represents another frequently recurring light‐limited condition where mixotrophy might be advantageous. There is literature evidence that in *Wolffia australiana*, a lower proportion of genes is expressed in a time‐of‐day fashion compared to other model species [26]. It could be hypothesized that this supports continuous growth, thereby offering a competitive advantage. Under such circumstances, DOC‐based mixotrophic and heterotrophic strategies likely compensate for reduced carbon assimilation, and these strategies appear widespread among duckweed species. In this review, we examine the prevalence of mixotrophy and heterotrophy in duckweeds and highlight their ecological and biotechnological significance.

## 4 Mixotrophic and Heterotrophic Duckweeds in Aquatic Environments

### 4.1 DOC as a Carbon Source for Photoautotrophic Duckweeds

Dissolved organic carbon, a water‐soluble fraction of organic matter, accounts for ∼80% of total organic matter in aquatic ecosystems [24, 27]. DOC includes amino acids, carbohydrates, lipids, polyols, humic substances, and small organic acids [24, 27]. Its concentrations may reach up to 300 mg/L in freshwater [28]. In the Haihe River basin (north China), DOC showed strong seasonality, with a spring median of 5.45 mg/L (range 0.76–40.04 mg/L) and autumn median of 4.56 mg/L (*p* < 0.01). DOC was positively related to Carlson's trophic state index, indicating higher DOC under more eutrophic conditions [29]. While much DOC originates from resident aquatic organisms, substantial inputs also derive from watersheds and riparian zones through anthropogenic activities [24]. Global warming exacerbates DOC dynamics by intensifying droughts and floods, leading to episodic spikes of up to 300 mg/L due to fluctuating water levels [30]. Additionally, ultraviolet (UV‐B) radiation and pH significantly influence DOC degradation, concentration, bioavailability, and relative endogenous versus exogenous origin [24]. Industrialization, agriculture, and urbanization have further elevated DOC concentrations, often exceeding natural background levels (∼0.1 mg/L) [30]. As a result, DOC represents a widely available carbon source that can sustain heterotrophic and mixotrophic growth in duckweeds.

### 4.2 Mixotrophy and Heterotrophy in Duckweeds

**FIGURE 2:**
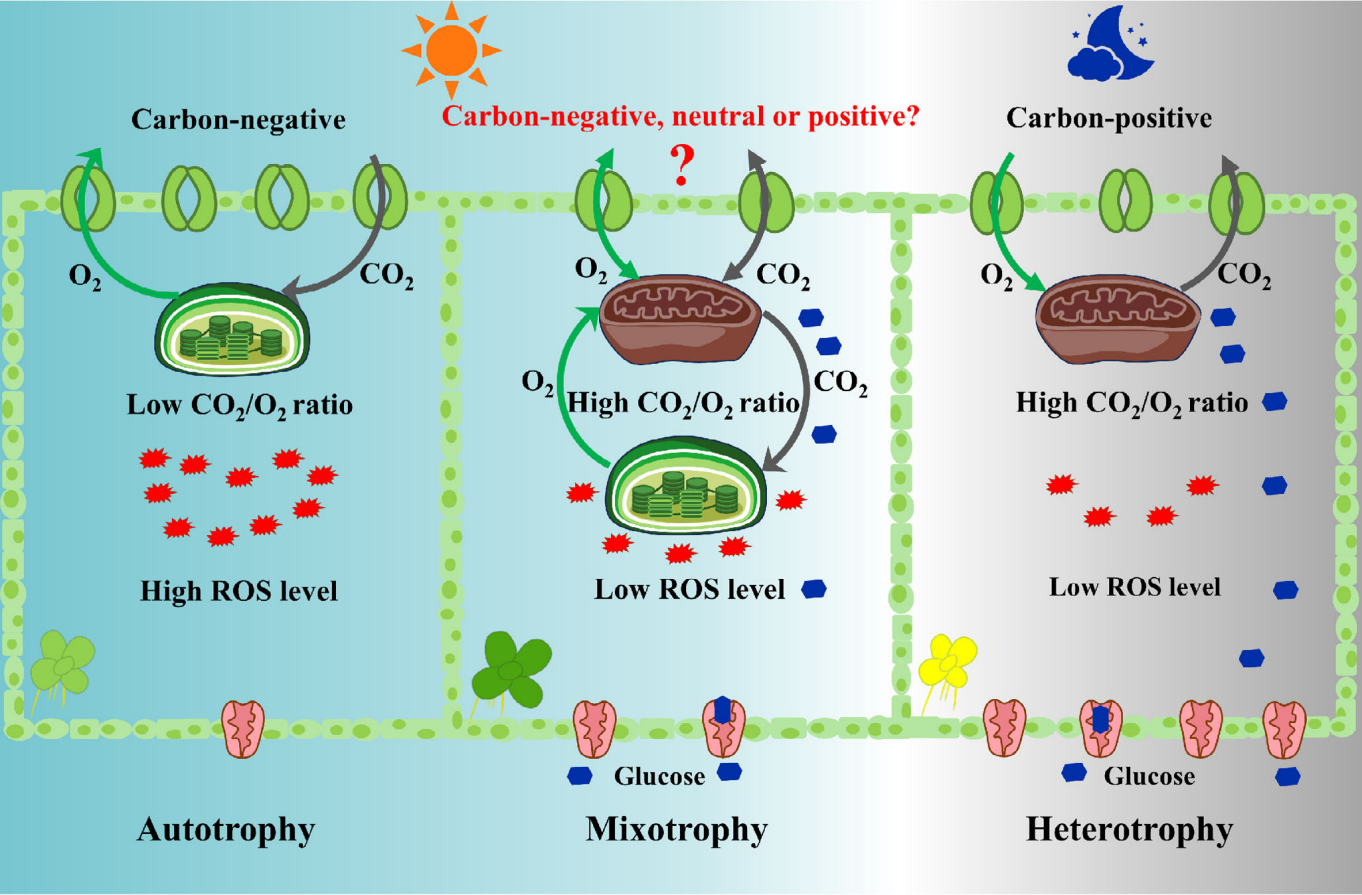
Mixotrophy and heterotrophy in duckweeds. Duckweeds, as C3 aquatic plants, fix carbon through photosynthesis by utilizing light energy and CO_2_ (autotrophy: carbon‐negative), and are also capable of absorbing dissolved organic carbon (DOC) from freshwater (heterotrophy: carbon‐positive). The simultaneous employment of photosynthesis and DOC assimilation defines mixotrophy; thus, depending on the relative balance between autotrophy and heterotrophy, mixotrophy may result in carbon‐negative, carbon‐neutral, or carbon‐positive outcomes.

Duckweeds acquire carbon by either the direct uptake of exogenous organic compounds in darkness, a process known as heterotrophy, which is carbon‐positive (carbon source), or through photosynthetic fixation of CO_2_ under illumination, referred to as photoautotrophy, which is carbon‐negative (carbon sink) (Figure 2) [5, 23]. Mixotrophy integrates these two pathways, forming a functional continuum between photoautotrophy and heterotrophy, and may be characterized as carbon‐negative, carbon‐neutral, or even carbon‐positive, depending on the relative contributions of autotrophic and heterotrophic processes (Figure 2).

Heterotrophic nutrition is a common feature in nature. Even primarily photoautotrophic plants exhibit heterotrophic metabolism in organs exposed to permanent or temporary darkness (e.g., seed germination, roots, rhizomes). This metabolic flexibility demonstrates that photoautotrophic organisms are preadapted to acquire organic matter when carbon sources shift from endogenous to exogenous [31]. Mixotrophy is a complex process that involves the uptake of organic carbon sources in different ways. Absorbotrophic mixotrophy refers to the acquisition of low‐molecular‐weight organic substances through active or passive transport across cell membranes. Biotrophic mixotrophy involves obtaining organic carbon from another living organism, either through parasitism or mutualism. Necrotrophic mixotrophy, in contrast, relies on organic carbon derived from prey that are killed, such as small crustaceans in the case of aquatic plants like *Utricularia* spp. [3]. In this review, we adopt the categorization of mixotrophy proposed by Selosse et al. [23]. We hypothesize that mixotrophic duckweeds primarily acquire organic carbon directly from freshwater, a process well documented in microalgae and cyanobacteria [32, 33].

### 4.3 Ecological and Biotechnological Significance of Mixotrophy in Duckweeds

Mixotrophy can serve as an alternative nutritional strategy under various stress conditions, particularly in environments with nutrient deficiencies [34]. Plants growing in low‐light habitats often rely on mixotrophy to meet their carbon demands. For example, the forest plant *Cephalanthera damasonium* adapts to shade through mixotrophy, obtaining organic nutrition from root‐associated fungi [35]. Similarly, carnivorous plants such as *Nepenthes* acquire organic phosphorus and nitrogen through mixotrophy in nutrient‐poor environments [35]. For duckweeds, mixotrophy is of major ecological significance because it enables them to become locally abundant by using additional organic matter, in contrast to the limitations of strict photoautotrophy or heterotrophy [36]. Mixotrophic duckweeds also play an important role in carbon cycling: by assimilating more carbon from aquatic ecosystems than strict photoautotrophs, they achieve greater biomass accumulation [5].

In freshwater environments, mixotrophic microalgae are most often found in oligotrophic systems where high DOC concentrations are coupled with low‐light conditions [37]. In such contexts, they play a critical role in carbon capture [38]. Given that organic carbon pollution favors mixotrophy and heterotrophy, duckweeds are well‐suited for wastewater treatment in carbon‐rich environments [39]. A growing consensus regarding the diversity of mixotrophy across microalgae, protists, and aquatic plants underscores its broader ecological importance [3].

High fluctuations in inorganic carbon and light availability in aquatic ecosystems likely impose strong selection pressures for the maintenance of metabolic flexibility [40]. The metabolic versatility of duckweeds offers valuable opportunities for biotechnological exploitation. Duckweeds have already been applied in food and animal feed, bioenergy, bioremediation, and antioxidant production [6]. Their trophic diversity should be further explored by cultivating them in organic carbon‐rich wastewaters or using inexpensive organic carbon sources [14]. Such strategies could significantly reduce the operational costs of duckweed biomass production while providing environmental benefits.

## 5 Old and New Evidence of Mixotrophy and Heterotrophy in Duckweeds

### 5.1 A Research Void in Duckweed Heterotrophy and Mixotrophy

Although early studies dating back to the 1930s [41, 42, 43] demonstrated the mixotrophic capacity of duckweeds, the physiological mechanisms and ecological boundaries of this trait have only recently been revisited in depth [5, 44]. First, photoautotrophy in aquatic plants appears to have been less investigated compared to land plants over the past three decades. Second, aquatic microorganisms‐especially heterotrophic microbes‐are highly efficient at metabolizing organic carbon [23], which may have led researchers to overlook the ability of duckweeds to compete effectively in freshwater ecosystems [45]. Nevertheless, the persistence of locally high DOC concentrations suggests that large amounts of organic carbon are indeed available for duckweeds, favoring mixotrophic strategies.

### 5.2 Arguments in Support of Mixotrophy and Heterotrophy in Duckweeds

Because low CO_2_ levels in aquatic environments often limit plant growth, an important consideration is that CO_2_ released from heterotrophic bacterial DOC respiration may enhance the photosynthetic performance and growth of photoautotrophic duckweeds [3]. To avoid such indirect effects and accurately assess the intrinsic mixotrophic capacity of duckweeds, axenic culture conditions are required. An important consideration is the role of associated microorganisms. In non‐axenic cultures, bacteria and fungi may compete with duckweeds for DOC, alter oxygen availability and medium chemistry, or release metabolites that either facilitate or inhibit duckweed growth [46]. Heterotrophic microbial respiration may indirectly stimulate photosynthesis through CO_2_ release, but microbial overgrowth under organic carbon supplementation may also impair duckweed performance and even cause frond decline or death [46]. Therefore, axenic systems are essential for demonstrating intrinsic duckweed mixotrophic capacity, whereas non‐axenic systems are necessary for understanding how this capacity is expressed under realistic ecological and wastewater conditions. The following examples provide strong evidence supporting mixotrophy and heterotrophy in several duckweed species.

As early as 1931, studies demonstrated that glucose and sucrose substantially stimulated the mixotrophic growth of duckweeds under low‐light conditions [43]. Subsequent experiments showed that glucose, sucrose, and fructose‐but not mannitol, maltose, glycerol, or ethanol‐significantly promoted the growth of *Spirodela* and *Lemna* species [47]. Hillman [48] further reported that *Lemna minor* did not respond to starch, lactose, arabinose, ribose, tartrate, succinate, or acetate as carbon sources. Collectively, earlier results indicated that sucrose enhanced the growth of all duckweed strains tested under light‐limited conditions, with optimal concentrations between 0.5% and 2.0% [43, 49, 50, 51]. The most detailed investigations of heterotrophy in duckweeds were conducted in *L. minor*, the first species successfully grown under heterotrophic conditions on a medium containing a gradient of yeast extract, casein hydrolysate, sucrose, and minerals [42]. These studies revealed that sucrose alone was insufficient to promote heterotrophic growth; likewise, yeast extract or casein hydrolysate without sucrose had no effect. Instead, all three organic components together, in combination with minerals, were essential for growth.

During the 1990s, it was reported that *L. minor* and *Landoltia punctata* exhibited higher growth rates when provided with sucrose or galactose as organic carbon sources under both light (mixotrophic) and dark (heterotrophic) conditions compared with strictly photoautotrophic controls [41, 52]. Earlier work, however, had shown that *L. minor* and *Wolffia brasiliensis* grew poorly with galactose [51]. Although many of these experiments used carbohydrate concentrations much higher than those found in natural freshwater ecosystems, they nevertheless demonstrated that duckweeds are capable of utilizing diverse organic carbon sources under both mixotrophic and heterotrophic conditions. Notably, an achlorophyllous mutant of *Lemna gibba* grown on sugar achieved growth rates comparable to wild‐type plants under light [51]. Similarly, Vidakovič‐Cifrek et al. [53] reported that sucrose (0.05–0.10%) increased the growth rate of *L. minor* under low‐light conditions. More recently, our studies showed that xylose, glucose, galactose, fructose, maltose, and sucrose all substantially enhanced the mixotrophic growth of *Spirodela polyrhiza* [14, 36, 54]. Except for xylose, these sugars also supported the heterotrophic growth of *S. polyrhiza* under sterile conditions [14].

The use of duckweeds in phytoremediation of organic carbon‐rich wastewaters provides further indirect evidence for their mixotrophic ability [39]. Over the past decades, duckweeds have been widely applied to remove nitrogen‐ and phosphorus‐containing organic pollutants from water [55]. They also significantly reduce the chemical oxygen demand (COD) of wastewater [39, 54]. While heterotrophic growth contributes to wastewater cleaning, particularly in ecological contexts where natural processes are prioritized, it is acknowledged that growth rates under strictly heterotrophic conditions may be too slow for rapid industrial biotechnological processing. Therefore, strategies often focus on mixotrophic conditions to balance efficiency and remediation capacity.

## 6 Reconsideration of Heterotrophy and Mixotrophy in Duckweed‐Favorable Physiology

Several physiological and structural traits of duckweeds support their ability to grow under mixotrophic and heterotrophic conditions. Their genomes contain an abundance of sugar transporter genes [44, 56], indicating the capacity to utilize a broad spectrum of organic carbon sources and adapt to variable environments. Duckweeds also have reduced or very thin cuticle, facilitating carbon and energy exchange with the aquatic environment [11, 57]. Furthermore, both their fronds and adventitious roots absorb water and nutrients, markedly increasing their surface area for exchange. As a result, organic compounds can be readily transferred from the extracellular to intracellular space via transporters [11, 37]. Unlike terrestrial plants, duckweeds do not face seasonal drought constraints, as their respiration is not controlled by stomata [3]. Collectively, these favorable physiological characteristics, combined with environmental selection pressures, confer significant advantages to mixotrophy over strict photoautotrophy or heterotrophy. By exploiting multiple carbon and energy sources, duckweeds enhance their survivability, facilitating adaptation to diverse niches and contributing to ecological and genetic diversity.

In land plants, mixotrophy is considered an evolutionarily stable equilibrium because heterotrophically acquired organic carbon is invested in vegetative growth, whereas photosynthetically fixed carbon is preferentially allocated to sexual reproduction [58]. This partitioning prevents a complete evolutionary shift toward heterotrophy [59]. In duckweeds, however, most carbon resources are allocated to vegetative reproduction, with sexual development largely reduced to favor rapid growth [60]. Thus, heterotrophy in duckweeds may represent an opportunity with minimal impact on overall fitness. A key question is whether the evolution of absolute heterotrophy, with a complete loss of photosynthesis, could occur in duckweeds [61]. Experiments with achlorophyllous *L. gibba* mutants demonstrate that such a transition is genetically feasible [51]. Nonetheless, the selective pressures in natural ecosystems do not appear strong enough to favor the total loss of photosynthesis compared with other adaptive traits. Data remain scarce, but future studies should investigate whether high‐DOC environments with limited CO_2_ and/or light could impose sufficient selective pressure for the emergence of obligate heterotrophy.

## 7 Favorable Conditions of Freshwater Ecosystems for Mixotrophy in Duckweeds

**FIGURE 3:**
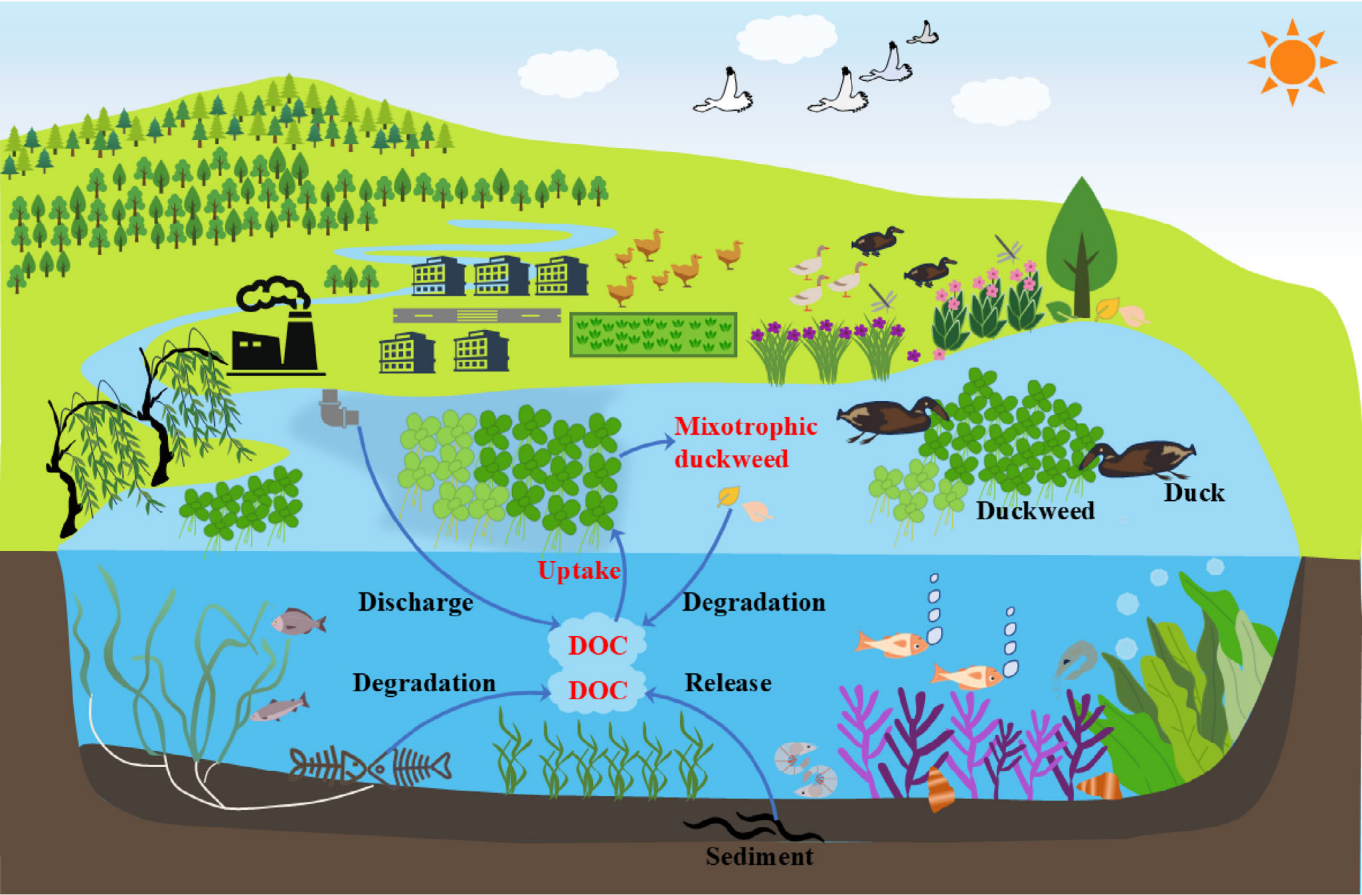
In freshwater environments, elevated concentrations of DOC‐resulting from biological activity (e.g., animals, plants, microorganisms) and anthropogenic sources, together with reduced light availability, serve as dual selective pressures that promote the development of mixotrophy in duckweeds.

Freshwater ecosystems inherently favor heterotrophy and mixotrophy in duckweeds (Figure 3). Their diverse trophic metabolisms represent a successful survival strategy in variable aquatic environments. In duckweeds, mixotrophy and heterotrophy are supported by several main factors. First, increased capacity to exchange substances with the aquatic environment due to the absence of cuticle and bark, and easy intercellular communication. Second, light‐limited stress conditions: depending on turbidity, depth, and shade, light intensity and quality vary considerably. Therefore, strictly photoautotrophic duckweeds are often subject to light limitations caused by shading, depth, and turbidity, which inhibit photosynthesis to varying degrees [3, 62]. Light availability is further restricted by suspended particles and humic substances, especially during extreme events such as algal blooms or floods [3]. Consequently, floating and submerged duckweeds (e.g., those along shaded banks, beneath emergent vegetation, or entire populations during flood‐induced turbidity) experience reductions in photosynthetic performance, which can be compensated by mixotrophy and heterotrophy. Duckweed fronds constantly produce new offspring fronds, which require some time to build up their own photosynthetic machinery and to acclimate to ambient conditions [46]. Hence, in a duckweed colony, there are both mature and developing fronds all the time with supposedly different metabolic contributions from autotrophy and heterotrophy. In this regard, mature fronds can be considered as carbon sources, while the younger fronds as rather carbon sinks which might also benefit from using external carbon sources [46]. Of course, besides ontogeny, the actual level of mixotrophy in a given frond is also under the influence of ambient conditions (e.g., DOC, CO_2_ and, light availability) [46, 63]. Seasonal droughts and anthropogenic activities also contribute significantly to elevated DOC levels [3]. Over the past decades, industrial and agricultural discharges have resulted in widespread eutrophication of freshwater ecosystems worldwide [37]. In this context, heterotrophy and mixotrophy confer strong evolutionary advantages over strict photoautotrophy, particularly during periods of high DOC concentrations or flooding [64].

Another important factor is the low availability of inorganic carbon in freshwater environments. Aquatic plants can use atmospheric CO_2_, dissolved CO_2_, and bicarbonate (HCO_3_^−^) as carbon sources [23]. In standing waters, limited CO_2_ diffusion strongly restricts submerged duckweed species such as *Lemna trisulca*, making organic carbon a valuable alternative. A recent study demonstrated that elevated CO_2_ concentrations (2%) markedly enhanced duckweed biomass accumulation [65]. Most data on duckweed mixotrophy are derived from floating species with aerial access to CO_2_. However, for fully submerged species restricted by CO_2_ diffusion and without access to the water surface, reliance on mixotrophy is highly probable.

## 8 Possible Mechanisms of Mixotrophy in Duckweeds

Because the surface area of plant cells is limited, mixotrophic cells must allocate membrane space to transport both organic and inorganic compounds. For efficiency, they must balance the investment in photosynthetic and respiratory components so that the benefits outweigh the costs [66]. These energetic costs may reduce the efficiency of organic and inorganic carbon utilization, potentially resulting in lower growth rates compared with strict phototrophs or heterotrophs [67]. However, our previous work demonstrated that the growth rate and biomass accumulation of mixotrophic duckweeds significantly exceeded the sum of strictly photoautotrophic and heterotrophic growth, indicating that mixotrophy is not a simple additive process but rather produces a “1 + 1 > 2” effect [5]. This suggests that the benefits of mixotrophy outweigh its high metabolic costs, likely through a synergistic “crosstalk” between photoautotrophic growth (chloroplasts) and heterotrophic growth (mitochondria), enabling efficient use of carbon and energy sources [5, 64]. Mixotrophic metabolic mechanisms have been more thoroughly studied in microalgae and cyanobacteria, which exhibit diverse trophic modes. Several regulatory mechanisms have been proposed: (1) photosynthesis provides reducing power and energy but does not fix CO_2_ for organic matter synthesis; (2) respiration is facilitated by O_2_ released from chloroplasts, while photosynthesis is stimulated by CO_2_ released from respiration; (3) mixotrophy suppresses photorespiration, reducing waste of energy and substrates; and (4) mixotrophy enhances antioxidant capacity [32, 33, 68, 69].

Our recent work further investigated the underlying mechanisms of mixotrophy in duckweeds [5]. We found that CO_2_‐concentrating mechanisms (CCM) and photosynthetic activity were suppressed in mixotrophic compared with photoautotrophic duckweeds, whereas respiratory and anaerobic fermentation activities were significantly enhanced. In addition, photorespiratory activity and oxidative damage were markedly reduced in mixotrophic duckweeds [5]. These findings suggest that intracellular CO_2_ levels are higher and O_2_ levels are lower in mixotrophic compared with photoautotrophic plants. We therefore hypothesize that CO_2_ produced by glucose metabolism diffuses into the chloroplasts and supplements the external CO_2_ required for photosynthesis. This reduces the dependence on environmental CO_2_ uptake, saving energy and supporting duckweed growth [70].

Although the simultaneous use of two carbon and energy sources provides a competitive advantage [67, 71], maintaining both photosynthetic (chloroplast) and respiratory (mitochondrial) systems is energetically costly. In higher plants, the energy required to maintain photosynthetic activity can account for up to ∼50% of the total energy budget [72]. Mixotrophy may mitigate this by reducing investment in photosynthetic apparatus and decreasing reliance on photosynthetic carbon fixation when exogenous organic carbon is available [73]. Thus, carbon fixation via photosynthesis is downregulated, while external organic carbon is used directly [40]. Moreover, photosynthesis and sugar metabolism are inverse reactions with respect to matter and energy. Excessive recycling of CO_2_ by chloroplasts can be wasteful; therefore, downregulation of photosynthesis in the presence of organic carbon is advantageous. Photorespiration under current atmospheric conditions leads to the loss of nearly 30% of carbohydrates fixed in C_3_ plants [74]. Consequently, the elevated intracellular CO_2_ and reduced O_2_ levels in mixotrophic duckweeds substantially suppress photorespiration, conserving energy for growth. Studies in microalgae have shown that they abandon mixotrophy at high CO_2_ concentrations, ceasing uptake of external organic carbon once CO_2_ is no longer growth‐limiting [75]. Similarly, current atmospheric CO_2_ concentrations are a limiting factor for duckweed photosynthesis [65]. Duckweeds therefore fulfill their carbon demand partly by absorbing organic carbon from freshwater. It remains essential to investigate whether duckweeds preferentially use CO_2_ or organic carbon, and to determine whether mixotrophy persists under elevated CO_2_ conditions.

**FIGURE 4:**
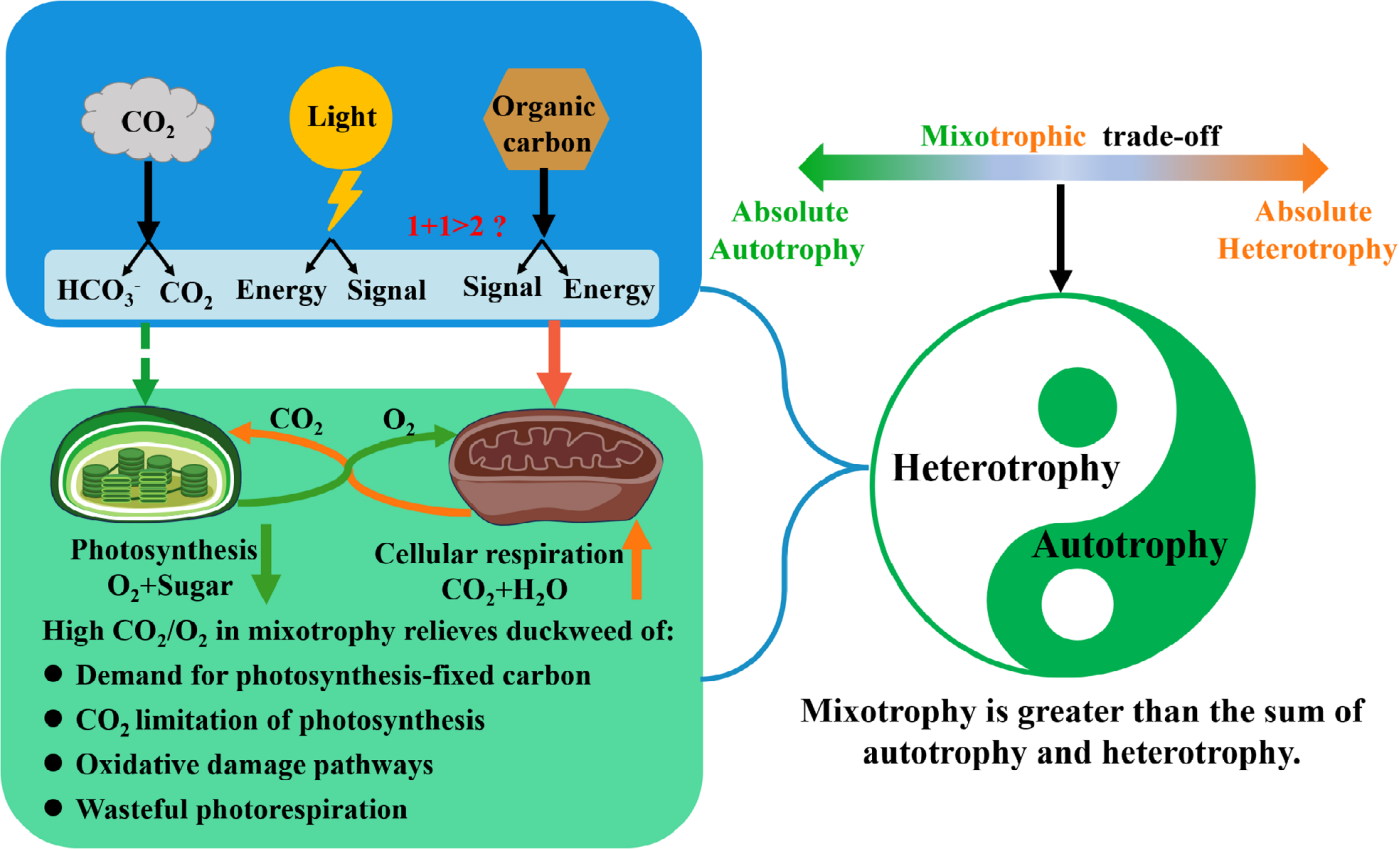
Possible mechanisms of mixotrophy in duckweeds. Mixotrophy reduces reliance on photosynthetic carbon fixation while alleviating chloroplast CO_2_ limitation through enhanced respiratory CO_2_ supply. High intracellular CO_2_ and low O_2_ concentrations suppress photorespiration and oxidative damage, thereby conserving energy. Consequently, mixotrophy results in greater biomass accumulation than the combined output of strict photoautotrophy and heterotrophy.

In conclusion, mixotrophy in duckweeds results from metabolic crosstalk between photosynthesis (photoautotrophy) and sugar metabolism (heterotrophy), whereby exogenous organic carbon can substitute for photosynthetic products to provide precursors for energy utilization. Compared with autotrophic duckweeds, mixotrophic plants downregulate CCM, photosynthesis, photorespiration, and oxidative pathways that consume energy or impair growth. This reduction in energy costs explains their superior biomass accumulation relative to the sum of autotrophy and heterotrophy [5] (Figure 4).

## 9 Concluding Remarks and Future Perspectives

Mixotrophic and heterotrophic nutritional strategies are pervasive in both terrestrial and aquatic ecosystems. However, compared with the extensive work on microalgae and land plants, the study of mixotrophy in aquatic higher plants remains limited. Moreover, researchers focusing on aquatic versus terrestrial models tend to work within distinct ecosystems and taxa, often overlooking broader connections. As a result, despite the ubiquity of mixotrophy and heterotrophy in the biosphere, many aspects of these strategies remain underexplored. The marked differences between aquatic environments and terrestrial ecosystems have also reinforced the underestimation of shared traits and questions. Mixotrophy exemplifies one such trait, suggesting that, beyond these differences, convergent mechanisms and constraints exist. Thus, there is an urgent need to deepen our understanding of mixotrophic mechanisms in aquatic ecosystems. Such knowledge may fundamentally reshape our view of aquatic plant function and wastewater depollution strategies. In this regard, duckweeds offer a valuable model system for elucidating mixotrophy across aquatic organisms.

Aquatic plants play a critical role in maintaining the proper functioning of aquatic ecosystems, where both inorganic and organic carbon are central to biogeochemical processes. Despite their coexistence with diverse carbon sources, many aquatic plants have never been systematically investigated for their mixotrophic and heterotrophic capacities. To date, most research has emphasized the physiological and metabolic aspects of these trophic strategies, whereas their roles in ecology, evolution, and development (Eco/evo/devo) remain poorly explored. Because mixotrophy integrates photoautotrophy and heterotrophy, the relative contributions of these two pathways to biomass accumulation may influence atmospheric carbon cycling in sustainable ways. It is therefore essential to study aquatic plant populations comprehensively from multiple perspectives to fully understand their ecological roles in aquatic systems.

## 10 Outstanding Questions

Which Environmental Factors Govern the Heterotrophy and Mixotrophy of Duckweeds? Could Anthropogenic Activities Such as Eutrophication Increase the Abundance of Heterotrophic and Mixotrophic Forms?

What role does respiration by external heterotrophic microbiota play in duckweed mixotrophy? Does it enhance photosynthesis by elevating intracellular CO_2_ availability? How does CO_2_ concentration regulate the mixotrophic strategy of duckweeds, and would mixotrophy disappear under sufficient CO_2_ supply?

What are the precise mechanisms coupling photoautotrophy and heterotrophy in mixotrophic duckweeds? To what extent does each pathway contribute to biomass accumulation, and which gene expression changes govern the transition between photoautotrophy and mixotrophy?

Since photosynthesis and glucose metabolism occur in distinct subcellular compartments‐including chloroplasts, cytoplasm, and mitochondria‐how do these processes interact to minimize losses of carbon and energy and achieve the observed “1 + 1 > 2” effect?

At the ecosystem scale, how do mixotrophic duckweeds acquire sufficient DOC to compete effectively with bacteria and fungi for organic carbon? Under what conditions might duckweeds cease competing with microbial heterotrophs?

Did mixotrophy evolve in the terrestrial ancestor of duckweeds, or was it acquired after their colonization of freshwater habitats? How often has mixotrophy evolved convergently in different aquatic phototrophs, and what are the similarities and differences in their underlying mechanisms?

What is the true extent of mixotrophy across duckweed taxa? Do species‐specific traits‐for example, floating fronds with or without roots, or fully submerged growth forms‐affect the degree of heterotrophy and mixotrophy?

## Author Contributions

Z.S. contributed to the original draft preparation. Y.C., F.L., X.Z., Q.H., and H.H. wrote, review and edited the manuscript. Funding acquisition was carried out by Z.S. and H.H. All authors have read and agreed to the published version of the manuscript.

## Conflicts of Interest

The authors declare no conflicts of interest.

## Data Availability

Data sharing not applicable to this article as no datasets were generated or analysed during the current study.
